# Editorial: Plasticity and flexibility in the parental brain

**DOI:** 10.3389/fnbeh.2024.1389613

**Published:** 2024-03-12

**Authors:** Natalia Uriarte, Mariana Pereira

**Affiliations:** ^1^Laboratorio de Neurociencias, Facultad de Ciencias, Universidad de la República, Montevideo, Uruguay; ^2^Department of Psychological & Brain Sciences, University of Massachusetts Amherst, Amherst, MA, United States

**Keywords:** maternal behavior, paternal behavior, family, behavioral flexibility, postpartum period

The present Research Topic features one mini review, one perspective, and a number of original research articles exploring various aspects of the neurobiological adaptations underlying caregiving decisions in health and disease, studied across animal models and humans.

Parenting in mammals is a transformative experience that requires a considerable amount of resources and energy that are essential to ensure the wellbeing of both the parents and their offspring. New parents undergo unique adaptations characterized by significant structural and functional plasticity in distributed brain circuits, which underlie processes of perception, motivation, affect, and cognition. The coordination among these processes relies on the action of various hormones and neurotransmitters, which facilitates contingent caregiving to meet the needs, developmental stage, and affect of the offspring.

This Research Topic highlights the flexibility of parenting, acknowledging how the maternal brain dynamically adapts to varying family situations. Hiura et al. examine how the presence of a parenting partner and acute stress influence caregiving in biparental prairie voles, throughout their offspring's development. The study offers an interesting perspective on how these factors collectively impact maternal behaviors, anxiety-like responses, and the vasopressinergic system. In this line of thought, Agrati and Uriarte advocate for the study of rodent maternal behavior in more challenging and ethologically relevant contexts than classical laboratory settings. In their perspective article, they discuss how the complexity surrounding postpartum mating and raising overlapping litters provides a unique and valuable model for investigating behavioral flexibility.

This Research Topic also considers the numerous neurobiological adaptations of the maternal brain, highlighting their role in facilitating supportive and sensitive parenting. In their minireview, Rivas et al. analyze the role of the hypocretinergic system in supporting caregiving activities by adjusting wakefulness and sleep patterns throughout the postpartum period. Hiraoka et al. explore changes in attentional bias toward infant crying with maternal experience and its relationship with caregiving intentions and depressive symptoms. Christensen et al. delve into the understudied effects of maternal age on adaptive mechanisms for sustaining cognitive regulation functions crucial to parenting.

This Research Topic also includes articles addressing advances in a less understood area: the transition into fatherhood and paternal programming effects on child development. Smiley et al. examine the neurobiological changes that lead virgin laboratory male mice to exhibit paternal behavior, providing valuable insights into the complex relationships between mating, prolactin and neurogenesis. Jones, De Braga et al. and Jones, Caccese et al., highlight emerging evidence that emphasizes the role of fathers' mental health during pregnancy and beyond on the neuroendocrine function and cognitive-behavioral outcomes of their children in middle childhood.

Together, the articles in this Research Topic provide innovative insights and advances our understanding of the brain mechanisms that enable parents to parent. We hope you enjoy reading this Research Topic and that it inspires new questions and future research. Studying the biological underpinnings of parental behavior from a fundamental perspective, in both animal models and humans, is crucial. This approach not only sheds light on the intricate mechanisms of parenthood but also highlights evolutionary continuities and variations across species. Understanding these biological underpinnings can inform interventions and policies aimed at supporting families and ensuring healthy development.

## Author contributions

NU: Writing – original draft, Writing – review & editing. MP: Writing – original draft, Writing – review & editing.

